# The value of moderate dose escalation for re-irradiation of recurrent or second primary head-and-neck cancer

**DOI:** 10.1186/s13014-020-01531-5

**Published:** 2020-04-16

**Authors:** Alexander Rühle, Tanja Sprave, Tobias Kalckreuth, Raluca Stoian, Erik Haehl, Constantinos Zamboglou, Roland Laszig, Andreas Knopf, Anca-Ligia Grosu, Nils H. Nicolay

**Affiliations:** 1 Department of Radiation Oncology, University of Freiburg – Medical Center, Faculty of Medicine, Robert-Koch-Str. 3, 79106 Freiburg, Germany; 2 German Cancer Consortium (DKTK) Partner Site Freiburg, German Cancer Research Centre (DKFZ), Heidelberg, Germany; 3 Department of Otorhinolaryngology, University of Freiburg – Medical Center, Faculty of Medicine, Freiburg, Germany

**Keywords:** Head-and-neck cancer, Head-and-neck squamous cell carcinoma (HNSCC), Recurrent head-and-neck cancer, Re-irradiation, Radiotherapy, Chemotherapy

## Abstract

**Background:**

Treatment for local and locoregional recurrence or second head-and-neck (H&N) cancers after previous radiotherapy is challenging, and re-irradiation carries a significantly increased risk for radiotherapy-related normal tissue toxicities and treatment failure due to a radioresistant tumor phenotype. Here, we analyzed re-irradiation management and outcomes in patients with recurrent or second primary H&N carcinoma using state-of-the-art diagnostic procedures and radiotherapy techniques.

**Methods:**

Between 2010 and 2019, 48 patients with recurrent or second primary H&N carcinoma received re-radiotherapy at the University of Freiburg Medical Center and were included in this study. Overall survival (OS) and progression-free survival (PFS) were calculated with the Kaplan-Meier method, and univariate Cox-regression analyses were performed to assess the effects of clinico-pathological factors on treatment outcomes. Acute and chronic treatment-related toxicities were quantified using the Common Terminology Criteria for Adverse Events (CTCAE v4.03).

**Results:**

Thirty-one patients (64.6%) received definitive and 17 (35.4%) adjuvant radiotherapy. Simultaneous chemotherapy was administered in 28 patients (58.3%) with cetuximab as the most commonly used systemic agent (*n* = 17, 60.7%). After a median time of 17 months (range 4 months to 176 months) between first and second radiotherapy, patients were re-irradiated with a median of 58.4 Gy and a treatment completion rate of 87.5% (*n* = 42). Median OS was 25 months with a 1-year OS amounting to 62.4%, and median PFS was 9 months with a 1-year PFS of 37.6%. Univariate analyses demonstrated that both a lower rT-status and a radiotherapy boost were associated with improved OS (*p* < 0.05). There was a trend towards superior OS for patients who received > 50 Gy (*p* = 0.091) and who completed the prescribed radiotherapy (*p* = 0.055). Five patients (10.4%) suffered from at least one grade 3 toxicities, while 9 patients (27.3%) experienced chronic higher-grade toxicities (≥ grade 3) with one (3.0%) grade 4 carotid blowout and one (3.0%) grade 4 osteoradionecrosis.

**Conclusion:**

Re-irradiation of recurrent or second primary H&N cancer with modern radiation techniques such as intensity-modulated radiotherapy resulted in promising survival rates with acceptable toxicities compared to historical cohorts. Increased re-irradiation doses, utilization of a radiotherapy boost and completion of the re-irradiation treatment were found to result in improved survival.

## Introduction

Treatment of local and locoregional recurrence or second head-and-neck (H&N) cancers after previous radiotherapy remains a challenge due to an increased risk of radiotherapy-related normal tissue toxicities and tumor radioresistance [1]. It has been reported that up to 30% of patients receiving definitive chemoradiotherapy for unresectable H&N cancer develop locoregional recurrences within 5 years, and long-term follow-up analyses from the RTOG 9501-trial revealed locoregional recurrence in up to 25% of patients treated with postoperative chemoradiotherapy for high-risk head-and-neck squamous cell carcinoma (HNSCC) [2, 3]. Based on the Radiation Therapy Oncology Group’s (RTOG) registry, about 23% of patients will develop a second primary cancer in the treatment region within 8 years after initial H&N cancer diagnosis. Surgery is considered an optimal curative treatment for medically operable patients with resectable recurrences and results in 5-year survival rates of up to 40% [4]. However, the prognosis for unresectable H&N carcinoma after initial radiotherapy is limited, and relatively poor survival rates have been observed after palliative chemotherapy [5]. Unfortunately, the GORTEC 98–03 trial, a randomized phase III-trial comparing chemo-re-irradiation with palliative chemotherapy, failed to accrue the intended patient population of 160 patients [6].

Compared to other tumor entities, there is increasing evidence for head-and-neck re-irradiation [7, 8]. Several retrospective series and two prospective RTOG phase II trials investigated the feasibility and oncological outcomes of chemoradiation for unresectable recurrent or second primary HNSCC after previous radiotherapy [9–14]. Different treatment protocols were used in the RTOG studies: While chemoradiation consisting of 60 Gy in 1.5 Gy twice-daily fractions and concomitant 5-fluorouracil/hydroxyurea were used in the older RTOG 9610 trial, twice-daily radiation in a split-course regime plus cisplatin/paclitaxel were applied in the RTOG 9911 trial [9, 10]. Although a distinct proportion of patients achieved long-term survival with these protocols, both the survival rates with 2-year OS rates of 15.2% (RTOG 9610) and 25.9% (RTOG 9911) as well as the toxicity rates with 8% treatment-related deaths in both studies were poor.

As analyses of patient cohorts using state-of-the-art diagnostic work-up with MRI and PET-CT as well as modern radiotherapy techniques are rare, we aimed to investigate patterns of management, oncological outcomes and toxicity analyses for re-irradiation of recurrent or second primary H&N cancer in a large retrospective single-center cohort.

## Methods

### Patients

All patients receiving re-irradiation for recurrent or second primary H&N cancer between 2010 and 2019 at the University of Freiburg Medical Center, Germany were included in this analysis. Electronic patient records were used to obtain demographic characteristics and clinical data, and pathological data including HPV and EBV status were taken from the pathology reports. Treatment decisions were based on multidisciplinary tumor board recommendations. Based on contrast-enhanced imaging and pathology reports, tumor classification was determined according to the 7th Edition of the UICC TNM Classification of Malignant Tumors. “Smokers” were defined as patients with a smoking history of ≥10 pack years. Development of a second primary H&N carcinoma was assumed if more than 60 months had passed since the index cancer diagnosis or if the second primary cancer occurred in a different region than the initial H&N cancer. This study was approved by the Independent Ethics Committee of the Medical Faculty, University of Freiburg (record no. 389/19).

### Diagnostic procedures and surgery

Primary work-up consisted of a detailed patient history, clinical examination, ultrasound of the neck and contrast-enhanced CT of the neck, thorax and abdomen. Whole-body FDG-PET-CT or MRI of the neck were performed based on availability and time period. For resectable recurrent or second primary H&N cancers, salvage surgery was the preferred treatment followed by adjuvant radiotherapy or chemoradiotherapy.

### Radiation treatment and chemotherapy

**Fig. 1:**
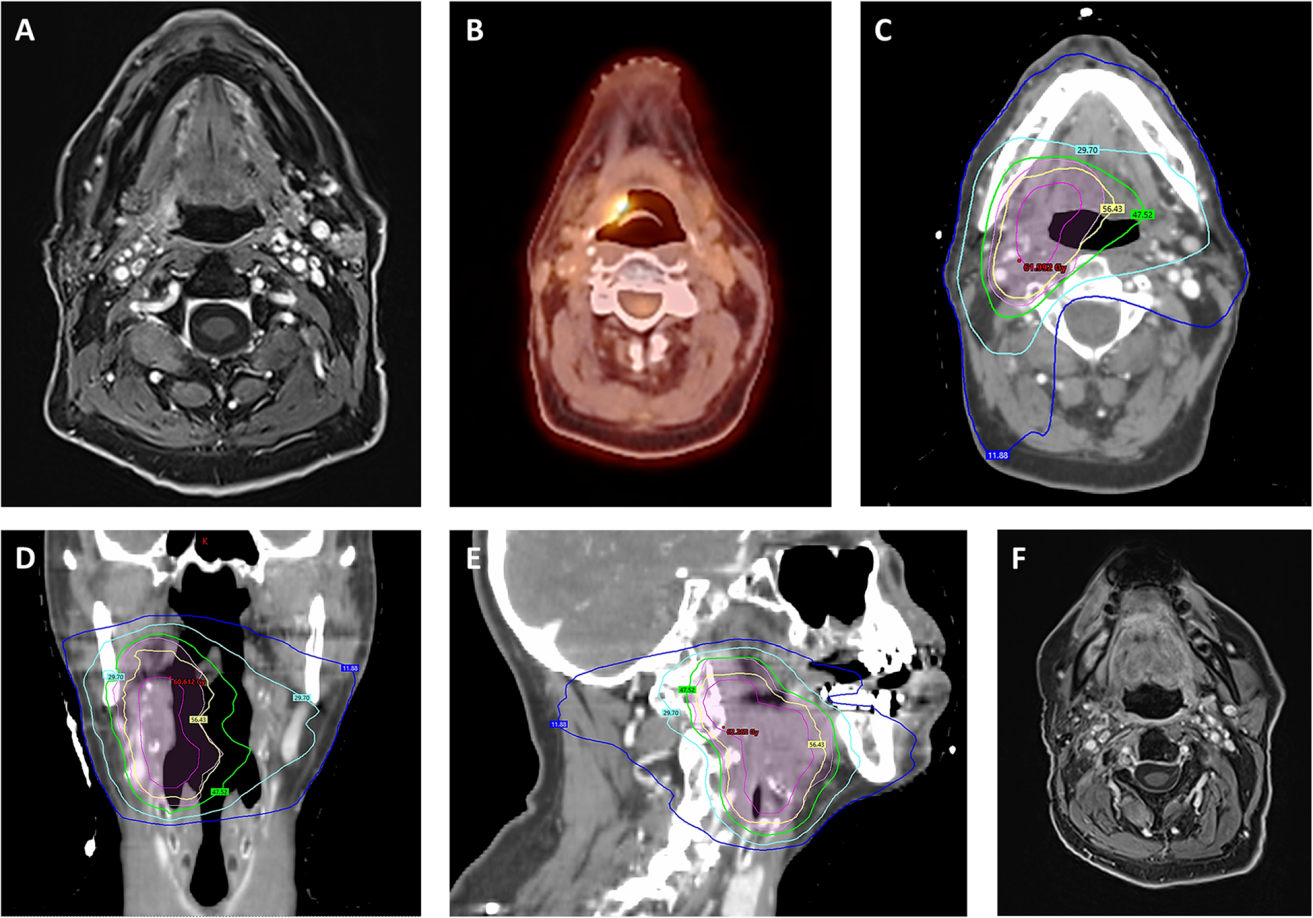
Re-irradiation for a recurrent oropharynx carcinoma in a 79-year-old male patient. The patient received chemoradiotherapy with 70 Gy for a HPV-positive cT4 cN2c cM0 oropharyngeal carcinoma (7th Edition of the UICC TNM classification) between March and May 2010. In July 2017, the patient developed a recurrent HPV-positive rcT2 rcN1 cM0 oropharyngeal carcinoma which was treated by re-irradiation with 52.2 Gy between September and October 2017. Initially, a total re-irradiation dose of 59.4 Gy was planned, but the treatment was discontinued after worsening of the patient’s general condition. Pretherapeutic MRI imaging (**a**) in July 2017 and FDG-PET-CT imaging (**b**) in August 2017 showing the recurrent rcT2 rcN1 cM0 oropharyngeal carcinoma on the right side. Based on the recommendations of the multidisciplinary tumor board, re-irradiation using IMRT was performed. Dose distribution of the IMRT plan is shown in an axial (**c**), coronal (**d**) and sagittal (**e**) scan image. PTV1 (pale pink) receiving 50.4 Gy in 28 fractions and PTV2 (pink) receiving 9 Gy in 5 fractions as sequential boost are shown as well as the lines for the 95%-isodose (yellow), 80%-isodose (green), 60%-isodose (cyan) and 20%-isodose (blue). The last MRI in November 2019 (**f**) showed no signs of recurrence

All patients received image-guided radiotherapy (IGRT), and the majority of patients were treated with intensity-modulated radiotherapy (IMRT) using either volumetric arc therapy (VMAT) or with helical tomotherapy (Fig. 1). Patients were immobilized with a thermoplastic head-neck mask. Re-irradiation volumes comprised gross tumor volumes (GTV) on imaging and an additional margin of 0.5–1 cm (CTV) based on anatomic localization and tumor size. In order to take organ motion and set up-errors into account, a planning target volume (PTV) margin of 0.5 cm was added to the CTV. In case a sequential radiotherapy boost was prescribed, the boost was administered in 5 fractions with 1.8 or 2 Gy single doses, leading to an additional boost dose of 9 Gy or 10 Gy, respectively. For simultaneous integrated boost (SIB) concepts, single doses to the boost volume ranged between 2 Gy and 2.5 Gy to a cumulative dose of around 60 Gy EQD2. Radiotherapy planning was conducted with Oncentra MasterPlan® (Nucletron BV, Veenendaal, the Netherlands) and Eclipse™ planning systems (Varian Medical Systems). Co-registration with the initial radiotherapy plan was performed, and isodoses of the initial irradiation were integrated in order to accurately assess the previous dose distribution. Dose constraints for organs at risk (OAR) were defined based on the previous exposure from the initial radiation treatment. In case of definitive radiotherapy or high-risk features in the postoperative setting such as close resection margins, incomplete resection (R1) or extracapsular spread (ECS), concomitant systemic therapy was administered.

### Outcome measures

Follow-up care consisting of clinical examinations and radiological imaging including ultrasound and cross-section imaging was performed every 3 months for the first 3 years and every 6 months for years 4 and 5. OS was defined as the interval from the start of re-irradiation to the last contact or death, and PFS was defined as the time from treatment initiation to locoregionally progressive disease or death. The record sections of the federal state authorities of Baden-Württemberg were contacted by the Comprehensive Cancer Center Freiburg in order to obtain missing survival data.

### Statistical analysis

OS and PFS were analyzed using the Kaplan-Meier method, and log-rank tests were performed to evaluate statistical significance. Univariate analyses of clinical and pathological parameters were performed separately with the Cox proportional hazard model. *P*-values< 0.05 were considered statistically significant. For all statistical analyses, IBM SPSS Statistics software version 22 (IBM, Armonk, NY, USA) was used.

## Results

### Patient characteristics and diagnostic work-up

Forty-eight patients received re-irradiation for recurrent or second primary H&N carcinoma between 2010 and 2019 and were included in this analysis. While 42 patients were reirradiated due to recurrence of the primary carcinoma, 6 patients received re-irradiation for a second primary H&N cancer. The study cohort consisted of predominantly male patients (*n* = 39, 81.3%) with a median age of 63 years (range 27–96 years) and a good performance status (*n* = 44, 91.7% ECOG 0–1) (Table 1). The majority of patients were smokers (*n* = 29, 60.4%), and the most common localizations of recurrent or second tumors were the cervical lymph nodes (*n* = 12, 25.0%) followed by the larynx (*n* = 10, 20.8%) and hypopharynx (*n* = 8, 16.7%). Distant metastases were present in 14 patients (29.2%) at the time of re-irradiation, and radiotherapy treatment was delivered with palliative intent for these patients. The most prevalent histology was squamous cell carcinoma (*n* = 38; 79.2%) with G2 (*n* = 27, 56.3%) as the most frequent grading. Of the 17 patients treated by adjuvant re-(chemo)radiotherapy, 8 patients had a positive resection margin. The majority of patients received PET-CT for re-staging and target volume definition prior to re-irradiation (*n* = 29, 60.4%).

**Table 1:** Patient characteristics including diagnostic work-up of patients treated by re-irradiation for recurrent or second primary H&N cancer in our institution between 2010 and 2019 (*n* = 48)

Variable		
**Age (median, range)**	63 (27–96)	
**Sex**	**n**	**%**
female	9	18.8
male	39	81.3
**ECOG**		
0	12	25.0
1	32	66.7
2	4	8.3
**Smoking**		
no	19	39.6
yes	29	60.4
**Localization recurrence**		
nasopharynx	3	6.3
oropharynx	4	8.3
hypopharynx	8	16.7
larynx	10	20.8
oral cavity	2	4.2
salivary gland	5	10.4
cervical lymph nodes	12	25.0
multi-level	3	6.3
others	1	2.1
**rT**		
0	23	47.9
1	4	8.3
2	2	4.2
3	5	10.4
4	14	29.2
**rN**		
0	27	56.3
1	10	20.8
2	9	18.8
3	2	4.2
**cM**		
0	34	70.8
1	14	29.2
**Histology**		
squamous cell carcinoma	38	79.2
adenocarcinoma	2	4.2
undifferentiated	2	4.2
others	6	12.5
**Grading**		
1	3	6.3
2	27	56.3
3	16	33.3
4	1	2.1
unknown	1	2.1
**Postoperative risk factors,*n*** **= 17**		
ECS	1	5.9
R+	8	47.1
R: close margin	2	11.8
Pn1	0	0.0
none of these above	6	35.3
**PET-CT**		
no	19	39.6
yes	29	60.4
**MRI**		
no	32	66.7
yes	16	33.3

### Radiotherapy characteristics

Thirty-one patients (64.6%) received definitive re-(chemo)radiotherapy, and 17 (35.4%) were treated with adjuvant re-(chemo)radiotherapy (Table 2). The median time between first and second radiotherapy were 17 months (range 4 to 176 months). Forty-two patients (87.5%) received the prescribed radiotherapy dose, and the most common reasons for premature treatment termination were the occurrence of acute radiotherapy-related toxicities or a deterioration of a patient’s performance status. About one in three patients (*n* = 15, 31.3%) received an additional simultaneously integrated or sequential radiation boost to the gross tumor or tumor bed. While a sequential boost was applied in 5 patients (10.4%) with a median boost dose of 10.8 Gy (range 9 to 16 Gy) in single doses of 1.8 Gy or 2.0 Gy, 10 patients (20.8%) were treated using a simultaneous integrated boost concept with a median radiation dose of 59.4 Gy. Neither the rate of patients receiving concomitant chemotherapy nor the rate of metastasized patients nor the radiotherapy completion status did significantly differ between the boost- and no-boost groups (*p* = 0.082 for chemotherapy, *p* = 0.346 for distant metastases, *p* = 0.077 for radiotherapy completion (chi-square-tests), supplementary Table 1). The median PTV volume was 105.1 cm^3^ (range 16.7 to 905.3 cm^3^), and the average re-irradiation dose was 59.4 Gy (58.4 Gy_EQD2_) after an initial median radiotherapy dose of 68.2 Gy (68.0 Gy_EQD2_), leading to a cumulative radiation dose of median 121.3 Gy (122.8 Gy_EQD2_). While the re-irradiation dose for the radiotherapy completion-group ranged at 59.4 Gy (58.4 Gy_EQD2_), patients who did not complete the prescribed course received a median dose of 27.0 Gy (27.45 Gy_EQD2_). The initially prescribed re-irradiation dose for the non-completion group also amounted to a median dose of 59.4 Gy (58.4 Gy _EQD2_).

**Table 2:** Treatment characteristics of re-irradiation for recurrent or second primary H&N cancer

Variable	Median (Range)	
**Time between 1st and 2nd radiotherapy**	17 months (4–176 months)	
**Initial radiation dose, EQD2 (α/β = 10)**	68.0 Gy (30.8 Gy – 72.0 Gy)	
**Re-irradiation dose, EQD2 (α/β = 10)**	58.4 Gy (3.7 Gy – 66.0 Gy)	
**Re-irradiation dose for RT completion group, EQD2 (α/β = 10)**	58.4 Gy (30.1 Gy – 66.0 Gy)	
**Re-irradiation dose for the RT non-completion group, EQD2 (α/β = 10)**	27.45 Gy (3.7 Gy – 47.8 Gy)	
**Intended re-irradiation dose for the non-completion group, EQD2 (α/β = 10)**	58.4 Gy (49.6 Gy − 66.0 Gy)	
**Cumulative radiation dose, EQD2 (α/β = 10)**	122.8 Gy (63.7 Gy – 132.0 Gy)	
**Cumulative radiation dose for the boost-group, EQD2 (α/β = 10)**	124.0 Gy (89.2 Gy – 132.0 Gy)	
**Cumulative radiation dose for the no boost-group, EQD2 (α/β = 10)**	119.1 Gy (63.7 Gy – 131.6 Gy)	
**PTV**	105.1 cm^3^ (16.7 cm^3^–905.3 cm^3^)	
**Cumulative D**_**max**_**spinal cord, EQD2 (α/β = 3)**	41.0 Gy (9.9 Gy – 74.8 Gy)	
	**n**	**%**
**Radiotherapy**		
definitive	31	64.6
adjuvant	17	35.4
**Completion radiotherapy**		
no	6	12.5
yes	42	87.5
**Boost**		
no boost	33	68.8
integrated boost	10	20.8
sequential boost	5	10.4
**Simultaneous chemotherapy**		
no	20	41.7
yes	28	58.3
**Chemotherapy,*n*** **= 28**		
cetuximab	17	60.7
cisplatin	8	28.6
cisplatin/5-fluorouracil	1	3.6
cisplatin/cetuximab	1	3.6
carboplatin	1	3.6

The median cumulative maximum dose (D_max_) to the spinal cord was 50.3 Gy (maximum 85.7 Gy) with an EQD2 of 41.0 Gy (α/β 3 Gy). Twenty-eight patients (58.3%) received concomitant systemic treatment with cetuximab as the most commonly used agent (60.7%). Other treatment regimens used for simultaneous chemotherapy were cisplatin (28.6%), cisplatin/5-fluorouracil (3.6%), cisplatin/cetuximab (3.6%) or carboplatin (3.6%).

### Patient outcomes

**Fig. 2:**
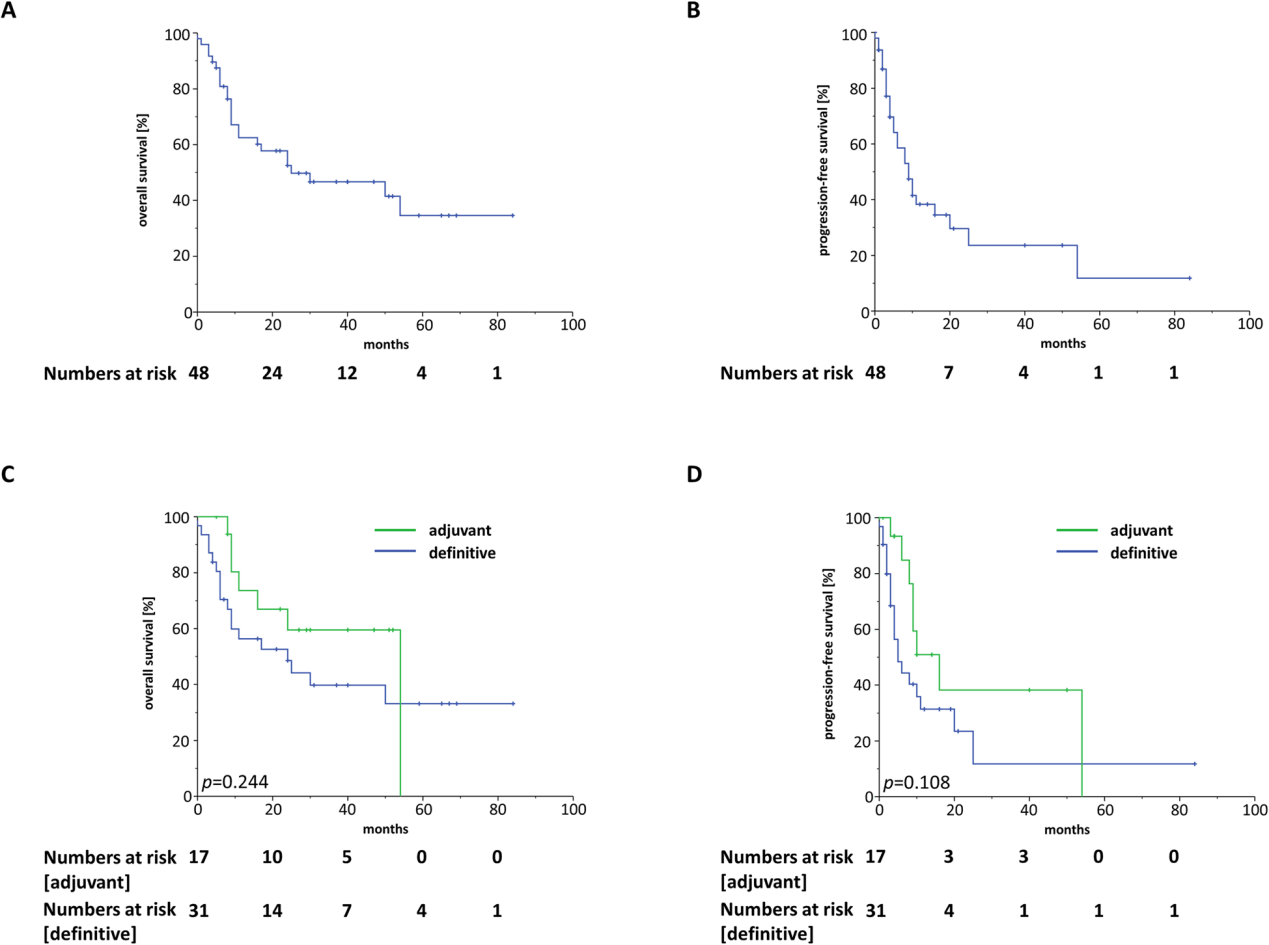
Kaplan-Meier curves regarding OS (**a**) and PFS (**b**) of patients treated by re-irradiation for recurrent or second primary H&N cancer between 2010 and 2019 (*n* = 48). OS (**c**) and PFS (**d**) of patients treated by definitive re-irradiation (*n* = 31) or adjuvant re-radiotherapy (*n* = 17). Log-rank tests were performed to compare different groups

Median OS in the patient cohort was 25 months, and 1-year, 2-year and 5-year OS were 62.4, 52.3 and 34.3%, respectively (Fig. 2a). Median PFS was 9 months, with the 1-year, 2-year and 5-year PFS amounting to 37.6, 28.8 and 11.5%, respectively (Fig. 2b). Both OS and PFS differed only insignificantly between patients treated with definitive or adjuvant re-irradiation (*p* = 0.244 for OS, *p* = 0.108 for PFS) (Fig. 2c and d).

**Fig. 3:**
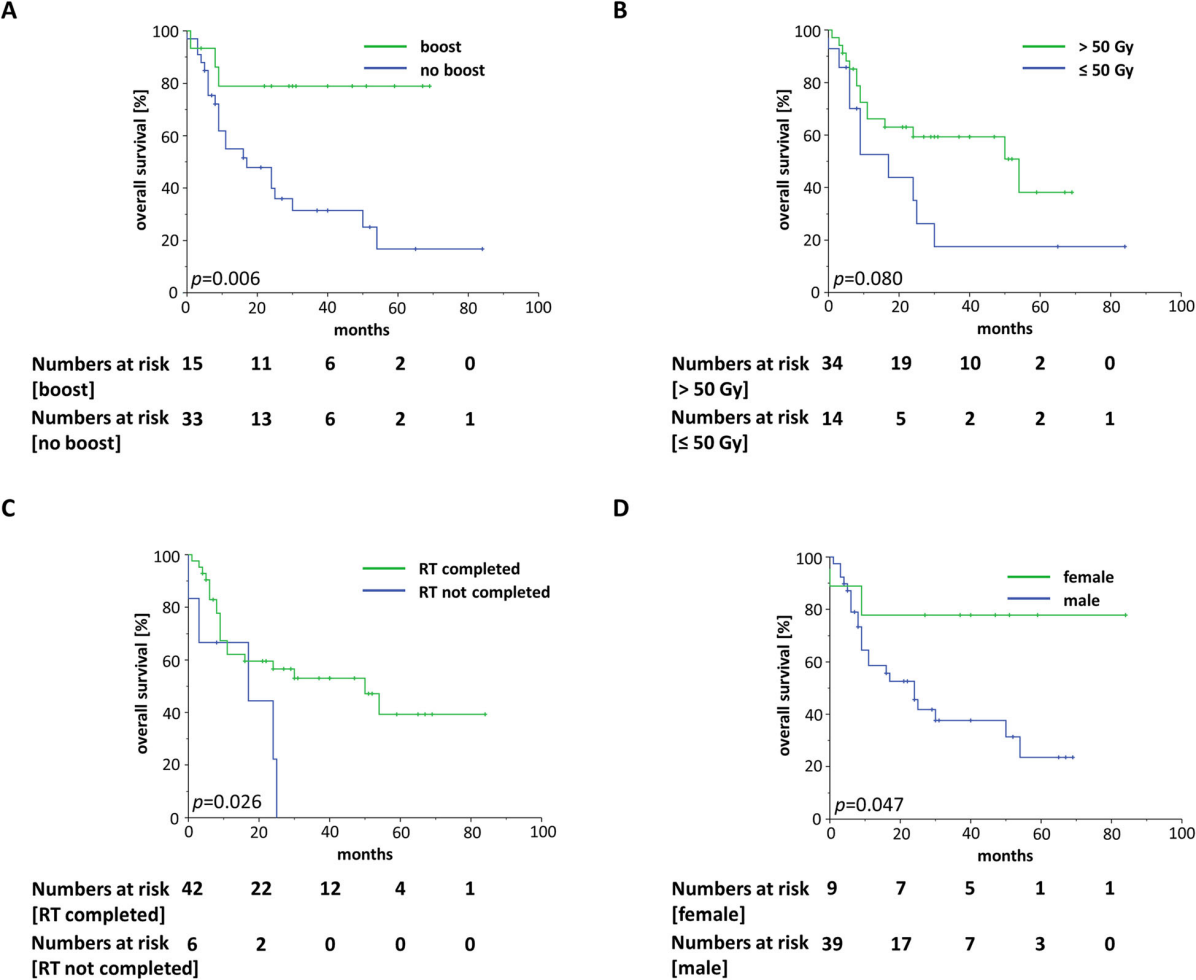
Kaplan-Meier curves showing OS according to several treatment-related and clinical parameters such as radiotherapy boost (**a**), total radiation dose (**b**), radiotherapy completion (**c**) and sex (**d**)

Separating Kaplan-Meier OS curves according to treatment-related factors showed that administration of a radiation boost and completion of the planned radiotherapy resulted in improved survival rates (*p* < 0.05, log-rank tests), whereby patients receiving increased re-irradiation doses of > 50 Gy showed a trend towards superior survival (*p* = 0.080) (Fig. 3a-c). Even if only patients who completed the prescribed course of re-radiotherapy were included in the survival analysis, patients receiving a radiotherapy boost still had superior OS rates (*p* = 0.017) (supplementary Figure 1). Additionally, male gender was found to be a risk factor for impaired OS (*p* < 0.05) (Fig. 3d). Univariate analyses showed that locally advanced tumors (rT3–4) were significantly associated with both reduced OS and PFS (OS: HR = 2.206, 95% CI 1.002–4.856, *p* < 0.05; PFS: HR = 2.940, 95% 1.351–6.397, *p* < 0.05) (Table 3). In line with the Kaplan-Meier survival data, there was a trend towards impaired OS in patients receiving reduced re-irradiation doses (≤ 50 Gy) (HR = 2.000, 95% CI 0.896–4.466, *p* = 0.091). Corresponding to the non-significant OS reduction for patients treated by radiation doses of ≤50 Gy, omission of a radiation boost resulted in significantly lower OS rates (HR = 4.544, 95% CI 1.354–15.256, *p* < 0.05).

**Table 3:** Univariate Cox-regression analysis regarding the effects of clinical and pathological parameters on OS and PFS

	OS			PFS		
Parameter	HR	CI 95%	***p***-value	HR	CI 95%	***p***-value
Age ≥ 65 / < 65 years	1.398	0.602–3.246	0.435	1.788	0.805–3.971	0.153
Gender male / female	3.832	0.898–16.352	0.070	1.646	0.564–4.800	0.361
ECOG 1–3 / ECOG 0	1.609	0.599–4.326	0.346	1.940	0.733–5.138	0.182
Smoker / non-smoker	1.603	0.690–3.722	0.272	1.743	0.786–3.867	0.172
rT3–4 / rT0–2	2.206	1.002–4.856	**0.049**	2.940	1.351–6.397	**0.007**
rN2–3 / rN0–1	0.680	0.255–1.814	0.441	0.636	0.256–1.580	0.330
M+ / M0	1.746	0.654–4.662	0.266	1.422	0.573–3.529	0.448
G3 / G1–2	0.882	0.396–1.966	0.759	1.072	0.494–2.327	0.860
No laryngeal carcinoma / laryngeal carcinoma	1.351	0.506–3.608	0.549	1.537	0.580–4.070	0.387
No PET-CT / PET-CT	0.880	0.388–1.998	0.760	0.837	0.385–1.817	0.652
No MRI / MRI	1.555	0.619–3.907	0.348	2.618	1.101–6.225	**0.029**
No boost / boost	4.544	1.354–15.256	**0.014**	2.330	0.803–6.758	0.119
≤ 50 Gy / > 50 Gy	2.000	0.896–4.466	0.091	1.752	0.801–3.833	0.160
Radiotherapy non-completed / completed	2.665	0.981–7.240	0.055	2.049	0.770–5.452	0.151
No chemotherapy / chemotherapy	1.019	0.448–2.321	0.964	0.801	0.368–1.742	0.576
Cisplatin / cetuximab	1.266	0.426–3.767	0.671	1.465	0.496–4.324	0.489

As other groups have reported superior survival results for laryngeal recurrence compared to other localizations, we compared laryngeal versus non-laryngeal recurrence and could not detect any significant survival differences (HR = 1.351, 95% CI 0.506–3.608, *p* = 0.549) [1]. Regarding the value of pre-therapeutic imaging for radiotherapy planning, we could not detect a PFS benefit for PET-CT but for MRI utilization (HR = 0.382, 95% CI 0.161–0.908, *p* < 0.05). Concomitant chemotherapy did not lead to improved OS or PFS rates (no chemotherapy vs. chemotherapy: HR = 1.019, 95% CI 0.448–2.321, *p* = 0.964), and there was no survival difference between patients receiving cisplatin or cetuximab (HR = 1.226, 95% CI 0.426–3.767, *p* = 0.671).

### Acute and chronic toxicities

Acute toxicities were assessed during radiotherapy as well as the first 90 days after completion of treatment and were quantified using National Cancer Institute-Common Terminology Criteria for Adverse Events (CTCAE v4.03). Dermatitis (68.8% grade 1–3), mucositis (60.4% grade 1–3) and xerostomia (60.4% grade 1–3) were the most common acute toxicities in our study population (details listed in Table 4). Overall, rates of grade 3 treatment-related acute toxicities were moderate and were observed for radiation dermatitis in 1 patient (2.1%) and for oral mucositis in 4 patients (8.3%). There were no acute grade 4 or 5 toxicities in our study. Regarding chronic treatment-related toxicities, we observed 2 grade 4 radiotherapy-related toxicities (6.1%), namely one carotid blowout and one osteoradionecrosis. Additionally, 4 patients (10.5%) suffered from grade 3 dysphagia, thereby the most common higher-grade chronic toxicity in our cohort. Other chronic grade 3 toxicities in our cohort were xerostomia, lymph edema and osteoradionecrosis.

**Table 4:** Acute and chronic radiotherapy-related toxicities according to the Common Terminology Criteria for Adverse Events (CTCAE v4.03)

	CTCAE grade					
Acute	0	1	2	3	4	5
Dermatitis	15	23	9	1	0	0
Dysphagia	37	10	1	0	0	0
Nausea and emesis	37	10	1	0	0	0
Mucositis	19	15	10	4	0	0
Xerostomia	19	25	4	0	0	0
Hoarseness	37	11	0	0	0	0
Dyspnea	45	3	0	0	0	0
Dysgeusia	24	21	3	0	0	0
Pain	24	21	3	0	0	0
**Chronic**	**0**	**1**	**2**	**3**	**4**	**5**
Pain	21	4	8	0	0	0
Lymph edema	27	3	2	1	0	0
Dysgeusia	23	7	3	0	0	0
Xerostomia	13	11	8	1	0	0
Dysphagia	20	3	6	4	0	0
Fistula	33	0	0	0	0	0
Carotid blowout	32	0	0	0	1	0
Osteoradionecrosis	31	0	0	1	1	0
Myelopathy	33	0	0	0	0	0

## Discussion

In our single-center analysis, we could demonstrate that patients receiving re-irradiation for recurrent or second primary H&N cancer with modern radiation techniques such as IGRT and typically IMRT exhibit favorable survival rates along with moderate higher-grade toxicities.

The promising survival rates associated with moderate toxicity in our study may be related to the utilization of modern radiation techniques such as IMRT in the majority of patients. Previous studies have shown that patients receiving conventional radiation techniques had higher rates of locoregional relapse compared to patients treated by IMRT [15]. Additionally, late higher-grade toxicities after chemoradiation for recurrent HNSCC were observed more frequently in patients treated by conventional radiotherapy [13]. Compared to other series reporting 2-year OS rates between 35 and 58% for re-irradiation of recurrent or second primary H&N cancer, the survival rates of our cohort with a 2-year OS of 52.3% are quite favorable [13–17].

Various studies have reported a relationship between increased total radiation dose and improved outcome in the event of re-irradiation [12, 18]. In a large multicentric retrospective analysis including 505 patients from 8 institutions, improved survival rates for patients with very good performance score after radiotherapy doses exceeding ≥66 Gy were observed in the definitive setting. Interestingly, radiotherapy doses were reported to have no significant impact on the outcomes for patients receiving adjuvant re-irradiation, if the doses ranged between 50 and 66 Gy postoperatively [18]. Due to the limited patient number in our study, we could not perform our dose escalation analyses separately for patients in the definitive or adjuvant re-irradiation setting. Our results pointed in the same direction and revealed a positive impact of an additional radiation boost and increased radiation dose regarding OS. Radiobiological analyses have demonstrated radioresistance induction in recurrent HNSCC, thus requiring higher radiation doses, alternative fractionation or radiosensitizing agents to achieve adequate tumor control rates [19, 20]. With increasing re-irradiation doses, the risk for significant and even life-threating toxicities is rising, and especially severe chronic adverse reactions are feared. While we observed acute grade 3 toxicities in about 10% of the entire patient cohort with no grade 4 or 5 toxicities, 27.3% of patients suffered from higher-grade chronic toxicities. The relatively low prevalence of higher-grade acute mucositis in our study may be related to smaller treatment volumes compared to initial definitive radiotherapy. Furthermore, older re-irradiation studies often used 5-fluorouracil as systemic agent, further increasing the mucositis severity of irradiated patients [11, 21, 22]. Recently, a competing risk nomogram was evaluated which can predict severe late toxicity at 2 years after re-irradiation in the head-and-neck region [23]. Radiotherapy dose for the first radiotherapy course, tumor site, organ dysfunction (pre-existing tracheostomy or feeding tube dependence), any surgery, patient’s age and the cause for re-irradiation (recurrent vs. second primary H&N cancer) were included in the risk nomogram. Interestingly, neither GTV volume, nor re-irradiation dose nor chemotherapy during the second radiotherapy course seemed to have an impact on the risk for severe late toxicities [23].

We detected one carotid blowout in our cohort consisting of 48 patients, which is consistent with previous re-irradiation studies reporting an incidence of 2.6% for this often lethal event [24]. While concomitant chemotherapy or previous salvage surgery did not increase the prevalence of carotid blow out in the systematic review by McDonald and colleagues, patients receiving accelerated hyperfractionation were at increased risk [24]. Local recurrence, an advanced T status and chemoradiotherapy were found to increase the risk of death related to carotid blowouts [25].

The GORTEC phase III randomized trial has evaluated the value of postoperative chemoradiation after salvage surgery of a recurrent or second primary HNSCC in a previously irradiated region [26]. The chemoradiation group received 60 Gy over 11 weeks combined with concomitant 5-fluorouracil and hydroxyurea, leading to a significant improvement of locoregional control and disease-free survival but not OS compared to patients without any adjuvant treatment. However, the addition of postoperative chemoradiation resulted in 39% grade 3–4 chronic toxicities compared to 10% in the observation group at 2 years after randomization. It is worth mentioning that 5 of 65 patients (7.7%) in the chemoradiation group died from treatment-related deaths. As there are no randomized trials comparing adjuvant re-irradiation with adjuvant chemoradiation after salvage resection, the value of concomitant chemotherapy for this cohort is unclear [27]. In our Cox regression analysis, simultaneous chemotherapy was not associated with improved survival with no differences between cetuximab and cisplatin. As our study is a retrospective analysis, omission of chemotherapy in unfit patients and presence of risk factors in patients receiving chemotherapy may bias the results of concomitant chemotherapy.

The localization of recurrent or second primary H&N carcinoma is known to influence the results of re-irradiation. Re-irradiation for recurrent nasopharynx or laryngeal cancer achieved superior local control rates compared to other anatomical sites such as oral cavity or hypopharynx [28]. Especially re-irradiation for recurrent nasopharynx carcinoma has demonstrated promising results with 5-year OS rates of 41% [29]. Due to the small patient numbers for nasopharynx carcinoma in our analysis, we did not compare the results between nasopharynx carcinoma and other localizations.

In the event of chemoradiation for recurrent or second primary H&N cancer, the type of simultaneous systemic therapy needs to be considered. The value of the epidermal growth factor receptor-inhibitor cetuximab in combination with stereotactic body radiotherapy (SBRT) for this patient cohort was investigated in some studies [30–33]. In the retrospective matched-cohort study of Heron and colleagues, combination treatment of SBRT and cetuximab achieved significantly higher local control rates as well as improved OS after 2 years than SBRT alone [30]. Besides its treatment efficacy, addition of cetuximab to SBRT was not associated with impaired patient-reported quality of life in comparison with SBRT alone [31]. However, another retrospective analysis regarding re-irradiation in combination with cetuximab for recurrent H&N cancer reported relatively poor oncological outcomes with a median OS of 7 months [33]. Randomized prospective head-to-head comparisons between cisplatin and cetuximab for re-irradiation in the head-and-neck region are lacking. In our study, there were no outcome differences between cisplatin and cetuximab; however, the favorable toxicity profile of cetuximab compared to cisplatin is an important criterion for this patient cohort with limited prognosis. Similarly to our results, Dornoff and coworkers examined the results of re-irradiation in the head-and-neck region combined with cetuximab or cisplatin and reported comparable oncological results [34].

Re-irradiation using protons or heavy ions have demonstrated promising results for recurrent or second primary H&N cancer [35–38]. In a large retrospective study including 229 H&N cancer patients treated with carbon ion re-irradiation, the authors reported about a median OS of 26.1 months which was comparable to the OS rates in our study population [39]. Due to the dosimetric advantages of proton or heavy ion re-irradiation in the head-and-neck region, sufficient radiation doses can be delivered to the recurrent or second primary H&N carcinoma, while critical adjacent normal tissues such as spinal cord, brainstem, optic nerves and optic chiasm can be spared. Compared to protons and photons, heavy ions such as carbon ions exhibit a higher linear energy transfer (LET) leading to a greater relative biological effectiveness (RBE). Despite the proven dosimetric advantages of proton or heavy ion re-irradiation, prospective trials including cost-effectiveness studies are lacking and have yet to demonstrate measurable clinical advantages.

There are various studies reporting the results regarding SBRT for re-irradiation of recurrent or second primary H&N cancer [30, 40–43]; however, whether SBRT is superior to other modern radiotherapy techniques such as IMRT is controversial. Vargo and colleagues conducted a retrospective multi-institutional analysis including 414 patients to compare SBRT with IMRT for re-irradiation in the head-and-neck region. Inverse probability of treatment weighting was used to control for baseline cohort differences. Whereby the unadjusted 2-year OS was superior in the IMRT-group (35.4% in the IMRT-group versus 16.3% in the SBRT-group), survival rates were found comparable after adjusting for baseline differences. However, acute grade ≥ 4 toxicity rates were significantly lower in the SBRT (0.5%) than in the IMRT group (5.1%) [31].

One of the most challenging decisions remains the identification of patients with recurrent or second primary H&N carcinoma who would benefit most from re-irradiation. Ward and colleagues analyzed 412 patients from 7 institutions who received re-irradiation for recurrent or second primary HNSCC and examined potential prognostic factors associated with improved survival. Using a recursive partitioning analysis, they identified 3 distinct subgroups differing significantly in their OS rates; these data may help to select suitable patients for re-irradiation [44]. Patients with a time interval > 2 years from the first treatment and a primary resection (class I), those with > 2 years from the first treatment but with unresected tumors or patients ≤2 years from the first therapy without feeding tube or tracheostomy dependence prior to re-irradiation (class II), or those ≤2 years from the previous treatment course with pre-irradiation feeding tube or tracheostomy dependence (class III). Whereby patients with more than 2 years in between radiation treatments and primary resections exhibited a 2-year OS of 61.9%, 2-year OS ranged at 40.0% for patients with shorter intervals between radiation courses or unresected tumors and only at 16.8% for patients with shorter intervals and dependence on feeding tubes or tracheostomy prior to re-irradiation. Therefore, alternative treatment strategies such as palliative chemotherapy may be considered for patients in the latter groups.

During the last decades, the knowledge about the normal tissue tolerance especially for the spinal cord after re-irradiation has significantly increased [45]. No radiation myelopathy after re-irradiation has been reported, when the spinal cord dose was limited to a cumulative dose of 72 Gy EQD2 (α/β 3 Gy; corresponding to a biologically effective dose of 120 Gy) [45]. In our study, the cumulative D_max_ to the spinal cord was 41 Gy (α/β 3 Gy), and hence, we did not observe any radiation-induced myelopathy in our cohort.

A strength of our single-center analysis is the fact that patients received modern image-guided and often intensity-modulated treatments. However, considering the retrospective nature of the study and the limited patient number, there are several limitations. The fact that some risk factors such as a short time interval between first and second radiotherapy were not associated with impaired survival in our study may be due to the small sample size. Additionally, retrospective analyses of treatment-related toxicities especially of chronic adverse reactions are complicated, as follow-up consultations were not performed by all patients especially during end-of-life phases, leading to underrepresentation of treatment-related toxicities. Especially chronic toxicities such as chondronecrosis or soft tissue fibrosis were not routinely assessed in our cohort and may be underrepresented, as other series reported significantly higher rates of these chronic normal tissue reactions after re-irradiation [46–48]. Although the median OS of 25 months was relatively satisfying in our study, the median PFS of 9 months underlines the importance of additional treatment modalities in order to improve both locoregional control and distant metastasis-free survival. A case report has reported promising results of repeated re-irradiation combined with checkpoint inhibitors for recurrent nasopharyngeal cancer [49]. A phase I/II-trial (NCT03317327) is currently investigating the results of re-irradiation (60 Gy with 1.5 Gy fractions twice daily) combined with the checkpoint inhibitor nivolumab for recurrent HNSCC. Other immunotherapy agents are currently studied in combination with re-irradiation for recurrent HNSCC.

In summary, out dataset showed promising results and moderate toxicity of re-irradiation for recurrent or second primary H&N cancer by using modern photon-based radiation treatment techniques, when compared to older prospective and retrospective re-irradiation series. A trend towards improved survival was observed after increased radiation dose, radiotherapy boost and radiotherapy completion, suggesting an important role of moderate dose escalation also in the recurrent treatment situation.

## Supplementary information

**Additional file 1: Table S1.** Administration of concomitant chemotherapy in dependence of a radiotherapy boost-concept. The usage of concomitant chemotherapy and the rate of metastasized patients did not differ between the boost- and no boost-group. *p* = 0.082 for chemotherapy usage, *p* = 0.346 for the cM-status, *p* = 0.077 for the radiotherapy completion status (chi-square-tests).

**Additional file 2: Figure S1.** Kaplan-Meier curves showing OS in comparison of a radiotherapy boost concept. Only patients who completed the prescribed course of radiotherapy were included in this analysis (*n* = 42). The *p-*value of the log-rank tests is indicated.

## Data Availability

The datasets used and analyzed during the current study are available from the corresponding author on reasonable request.
