# Supplementary material for: The value of moderate dose escalation for re-irradiation of recurrent or second primary head-and-neck cancer
Source: Radiat Oncol. 2020 Apr 16;15:81. doi: 10.1186/s13014-020-01531-5 (PMC7164259; doi:10.1186/s13014-020-01531-5)
Supplement: Supplementary file 1 — Additional file 1: Table S1. Administration of concomitant chemotherapy in dependence of a radiotherapy boost-concept. The usage of concomitant chemotherapy and the rate of metastasized patients did not differ between the boost- and no boost-group. p = 0.082 for chemotherapy usage, p = 0.346 for the cM-status, p = 0.077 for the radiotherapy completion status (chi-square-tests). [file 13014_2020_1531_MOESM1_ESM.docx]

Supplementary table 1: Administration of concomitant chemotherapy in dependence of a radiotherapy boost-concept. The usage of concomitant chemotherapy and the rate of metastasized patients did not differ between the boost- and no boost-group. p=0.082 for chemotherapy usage, p=0.346 for the cM-status, p=0.077 for the radiotherapy completion status (chi-square-tests).

|  | **Concomitant chemotherapy** | **No concomitant Chemotherapy** | **cM0** | **cM1** | **Radiotherapy completed** | **Radiotherapy non-completed** |
| --- | --- | --- | --- | --- | --- | --- |
| **Radiotherapy boost** | 6 | 9 | 12 | 3 | 15 | 0 |
| **No radiotherapy boost** | 22 | 11 | 22 | 11 | 27 | 6 |
