# Supplementary figures and images for: The value of moderate dose escalation for re-irradiation of recurrent or second primary head-and-neck cancer
Source: Radiat Oncol. 2020 Apr 16;15:81. doi: 10.1186/s13014-020-01531-5 (PMC7164259; doi:10.1186/s13014-020-01531-5)

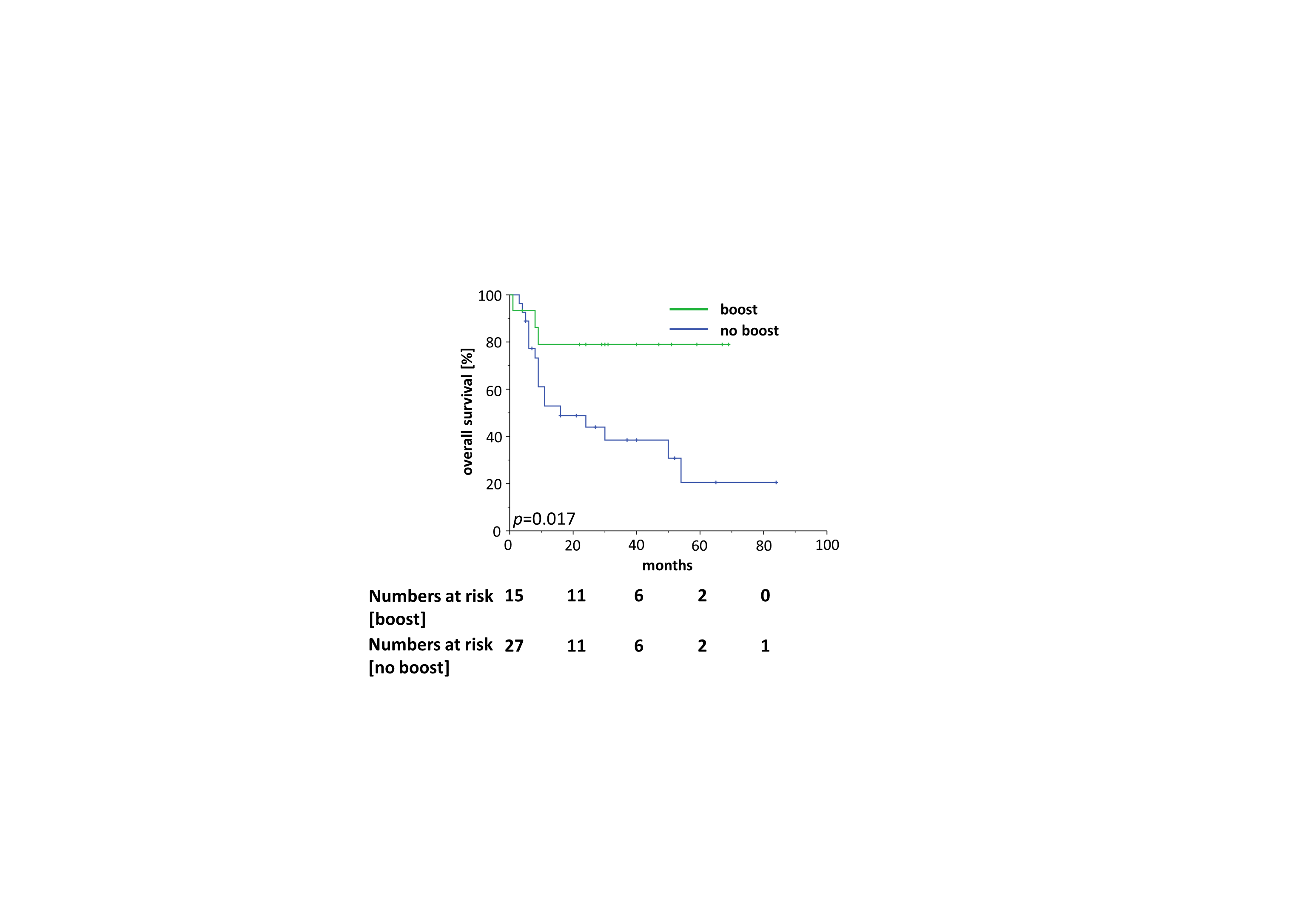

Supplement: Supplementary file 2 — Additional file 2: Figure S1. Kaplan-Meier curves showing OS in comparison of a radiotherapy boost concept. Only patients who completed the prescribed course of radiotherapy were included in this analysis (n = 42). The p-value of the log-rank tests is indicated. [file 13014_2020_1531_MOESM2_ESM.tif]
